# Analysis of the Molecular Diversity Among *Cronobacter* Species Isolated From Filth Flies Using Targeted PCR, Pan Genomic DNA Microarray, and Whole Genome Sequencing Analyses

**DOI:** 10.3389/fmicb.2020.561204

**Published:** 2020-09-25

**Authors:** Hyein Jang, Hannah R. Chase, Jayanthi Gangiredla, Christopher J. Grim, Isha R. Patel, Mahendra H. Kothary, Scott A. Jackson, Mark K. Mammel, Laurenda Carter, Flavia Negrete, Samantha Finkelstein, Leah Weinstein, QiongQiong Yan, Carol Iversen, Franco Pagotto, Roger Stephan, Angelika Lehner, Athmanya K. Eshwar, Seamus Fanning, Jeffery Farber, Gopal R. Gopinath, Ben D. Tall, Monica Pava-Ripoll

**Affiliations:** ^1^Center of Food Safety and Applied Nutrition, U. S. Food and Drug Administration, Laurel, MD, United States; ^2^WHO Collaborating Centre for Cronobacter, University College Dublin, Dublin, Ireland; ^3^UCD Centre for Food Safety, School of Public Health, Physiotherapy and Population Science, University College Dublin, Dublin, Ireland; ^4^Food Directorate, Bureau of Microbial Hazards, Health Canada, Ottawa, ON, Canada; ^5^Institute for Food Safety and Hygiene, University of Zürich, Zurich, Switzerland; ^6^Department of Food Science, University of Guelph, Guelph, ON, Canada; ^7^Center of Food Safety and Applied Nutrition, U. S. Food & Drug Administration, College Park, MD, United States

**Keywords:** *Cronobacter*, microarray, phylogenetic analysis, whole-genome sequencing, flies as insects pests

## Abstract

*Cronobacter* species are opportunistic pathogens capable of causing life-threatening infections in humans, with serious complications arising in neonates, infants, immuno-compromised individuals, and elderly adults. The genus is comprised of seven species: *Cronobacter sakazakii, Cronobacter malonaticus, Cronobacter turicensis, Cronobacter muytjensii, Cronobacter dublinensis, Cronobacter universalis*, and *Cronobacter condimenti*. Despite a multiplicity of genomic data for the genus, little is known about likely transmission vectors. Using DNA microarray analysis, in parallel with whole genome sequencing, and targeted PCR analyses, the total gene content of two *C. malonaticus*, three *C. turicensis*, and 14 *C. sakazaki* isolated from various filth flies was assessed. Phylogenetic relatedness among these and other strains obtained during surveillance and outbreak investigations were comparatively assessed. Specifically, microarray analysis (MA) demonstrated its utility to cluster strains according to species-specific and sequence type (ST) phylogenetic relatedness, and that the fly strains clustered among strains obtained from clinical, food and environmental sources from United States, Europe, and Southeast Asia. This combinatorial approach was useful in data mining for virulence factor genes, and phage genes and gene clusters. In addition, results of plasmidotyping were in agreement with the species identity for each strain as determined by species-specific PCR assays, MA, and whole genome sequencing. Microarray and BLAST analyses of *Cronobacter* fly sequence datasets were corroborative and showed that the presence and absence of virulence factors followed species and ST evolutionary lines even though such genes were orthologous. Additionally, zebrafish infectivity studies showed that these pathotypes were as virulent to zebrafish embryos as other clinical strains. In summary, these findings support a striking phylogeny amongst fly, clinical, and surveillance strains isolated during 2010–2015, suggesting that flies are capable vectors for transmission of virulent *Cronobacter* spp.; they continue to circulate among United States and European populations, environments, and that this “pattern of circulation” has continued over decades.

## Introduction

*Cronobacter* species are opportunistic foodborne pathogens that have gained attention in both research and clinical areas for their ability to cause fatal infections including meningitis, septicemia, necrotizing enterocolitis, and pneumonia in neonates (infants of 28 days or younger) and infants (Iversen and Forsythe, 2003; Yan et al., 2012; Jaradat et al., 2014; Patrick et al., 2014; Tall et al., 2014). *Cronobacter* species also cause septicemia, pneumonia, wound, and urinary tract infections in vulnerable adults (Iversen and Forsythe, 2003; Gosney et al., 2006; Holý et al., 2014; Patrick et al., 2014; Alsonosi et al., 2015). Infantile infections have been epidemiologically linked to consumption of intrinsically- and extrinsically contaminated lots of reconstituted powdered infant formula (PIF) (Noriega et al., 1990; Himelright et al., 2002; Henry and Fouladkhah, 2019). Furthermore, *Cronobacter* species have been found associated with a variety of other foods such as dried spices, nuts, ready to eat and frozen vegetables, retail foods, and relevant ecological niches such as food production environments and farms, from where they can re-distribute and contaminate finished foods posing a risk to susceptible consumers and widening the scope of public health concerns (Iversen and Forsythe, 2003; El-Sharoud et al., 2008; Osaili et al., 2009; Brandão et al., 2017; Jang et al., 2018a,b; Vasconcellos et al., 2018; Silva et al., 2019; Jang et al., 2020).

Neonates are susceptible to invasive infection with *Cronobacter*, defined here as a culture-positive infection emerging from a case(s) of septicemia or meningitis infections. These cases often lead to poor patient outcomes with subsequent development of chronic neurological sequelae such as hydrocephalus, permanent neurological damage and disabilities resulting in life-long health challenges or death. The reported estimated mortality rate for this age group is ∼27% (Bowen and Braden, 2006; Friedemann, 2009; Jason, 2012).

*Cronobacter sakazakii, Cronobacter malonaticus*, and *Cronobacter turicensis* are considered to be the primary pathogenic species which cause the majority of severe illnesses (Yan et al., 2012; Jaradat et al., 2014; Tall et al., 2019). Other species of *Cronobacter* include *Cronobacter universalis, Cronobacter condimenti, Cronobacter muytjensii*, and *Cronobacter dublinensis*, which, except for *C. condimenti*, have been known to cause a variety of infections in humans (Iversen et al., 2008; Joseph et al., 2012a). Surveillance data obtained by analyzing hospital records through U.S. FoodNet suggest that there is a higher percentage of *Cronobacter* infections observed in adults than in infants (Patrick et al., 2014). Similar findings reported by Holý et al. (2014) and Alsonosi et al. (2015) from hospitalized Czech Republic patients showed that *Cronobacter* infections in adults were more common than that found in infants and that *C. sakazakii* sequence type (ST)4 and *C. malonaticus* ST7 were the primary pathogens causing adult infections. Moreover, Jason (2012) showed that ∼8% (7/82) of infected infants with invasive disease consumed breast milk without supplementation with PIF or human milk fortifiers prior to onset of illness. Furthermore, reports by Bowen et al. (2017), Sundararajan et al. (2018), and McMullan et al. (2018) support these findings suggesting that infants are now being infected through contaminated expressed breast milk and nursing. Taken together, these results suggest that other unidentified sources or vectors may be involved in the transmission of *Cronobacter*, specifically those infections associated with adults. Thus far, these sources and vectors have been difficult to identify.

Insects, such as filth flies, have been recognized as a potential and common vector for *Cronobacter* and two other foodborne pathogens, *Salmonella* spp. and *Listeria monocytogenes* (Hamilton et al., 2003; Mramba et al., 2006, 2007; Pava-Ripoll et al., 2012, 2015b). Pava-Ripoll et al. (2012) showed that filth flies can harbor these foodborne pathogens on both their external body surfaces and within their alimentary canals. These authors also determined that 14% of filth flies randomly collected from dumpsters outside of urban restaurants were positive for *Cronobacter* both internally and/or externally, demonstrating that these flies can serve as reservoir and vector for these pathogens (Pava-Ripoll et al., 2012).

Several studies have applied sequenced-based methods such as a pan-genomic DNA microarray to identify and characterize strains obtained through surveillance activities (Tall et al., 2015; Yan et al., 2015b; Chase et al., 2017; Kothary et al., 2017; Jang et al., 2018a,b). These studies showed that microarray analysis could correctly assess the phylogenetic relatedness among persistant *Cronobacter* strains obtained from PIF manufacturing facilities, among strains isolated from plant-origin foods and showed that clinically relevant strains were phylogenetically related to these plant-associated strains, and helped to understand the nucleotide divergence of outer membrane proteins captured within outer membrane vessicles. The microarray was also able to accurately assess each strain’s identity, could differentiate *Cronobacter* species from phylogenetically related species, and was a useful tool to assess phylogenetic relatedness and gene content among strains.

In this paper, we explore the likely possibility that strains associated with filth flies are related to strains obtained from other sources and this information supports the hypothesis that flies may serve as vectors for clinically relevant *Cronobacter.* The results presented in this study suggests that filth fly isolates are phylogenetically related to *Cronobacter* strains recovered from clinical, food, and environmental sources using the above described pan genomic microarray in parallel with whole genome sequencing and targeted PCR analyses. Virulence of a subset of strains was assessed using a recently described zebrafish embryo model of infection (Fehr et al., 2015; Eshwar et al., 2016). Lastly, the results from this study demonstrate the usefulness of this parallel next generation sequencing approach coupled with virulence assessment of strains as discriminatory characterization and identification tools for public health and source attribution. These pathotypes were as virulent to zebrafish embryos as other clinical strains; and that they continue to circulate among the United States and European populations, environments, and filth flies.

## Materials and Methods

### Isolation of *Cronobacter* From Filth Flies

*Cronobacter* strains shown in Table 1 were obtained from the external surfaces and alimentary canals of wild caught adult filth flies as follows: thirteen strains were isolated from ten *Musca domestica* (housefly), two strains from one *Sarcophaga haemorrhoidalis* (red-tailed flesh fly), two strains from one *Lucilia cuprina* (blowfly), one strain from a *Lucilia sericata* (common green bottle fly), and another strain from an adult muscoide fly belonging to the family *Anthomyiidae* (*Anthomyiidae* are a large and diverse family of *Muscoidea* flies). Strains were isolated using the method described by Pava-Ripoll et al. (2012, 2015b). All strains are available upon request to Dr. Ben Tall at CFSAN, U.S. FDA.

**TABLE 1:** *Cronobacter* strain and species IDs*, filth fly host species and site, *Cronobacter* serotype, and MLST sequence types of strains isolated during 2012 from various species of filth flies which were investigated in this study.

Strain ID	*Cronobacter* species	Filth fly species, sample type	Serotype	Sequence type	Accession number
Md25g^#^	*C. malonaticus*	*Musca domestica*, house fly, alimentary canal	Cmal O:2	7	MSAC00000000
Md99g	*C. malonaticus*	*M. domestica*, alimentary canal	Cmal O:1	60	MSAF00000000
Sh41g	*C. turicensis*	*Sarcophaga haemorrhoidalis*, red-tailed flesh fly, alimentary canal	Ctur UKN	569	MRZS00000000
Sh41s	*C. turicensis*	*S. haemorrhoidalis*, surface	Ctur UKN	569	MSAG00000000
Md1sN	*C. turicensis*	*M. domestica*, surface	UKN	519	VOEL00000000
Md1g	*C. sakazakii*	*M. domestica*, alimentary canal	Csak O:2	4	MSAH00000000
Md27gN	*C. sakazakii*	*M. domestica*, alimentary canal	Csak UKN	93	VOEK00000000
Md33g	*C. sakazakii*	*M. domestica*, alimentary canal	Csak O:1	8	MSAI00000000
Md33s^#^	*C. sakazakii*	*M. domestica*, surface	Csak O:1	8	MRXC00000000
Md35s	*C. sakazakii*	*M. domestica*, alimentary canal	Csak UKN	8	MRXD00000000
Md40g	*C. sakazakii*	*M. domestica*, alimentary canal	Csak O:2	8	MRXE00000000
Md6g	*C. sakazakii*	*M. domestica*, alimentary canal	Csak O:3	4	MRXB00000000
Md70g	*C. sakazakii*	*M. domestica*, alimentary canal	Csak O:2	4	MRXG00000000
Anth48g	*C. sakazakii*	*Anthomyiidae* spp., alimentary canal	Csak UKN	221	MRXF00000000
Md5s	*C. sakazakii*	*M. domestica*, surface	Csak O:3	4	MRWZ00000000
Md5g	*C. sakazakii*	*M. domestica*, alimentary canal	Csak O:2	4	MRXA00000000
Lc10s	*C. sakazakii*	*Lucilia cuprina*, blowfly, surface	Csak O:3	4	NDXD00000000
Lc10g	*C. sakazakii*	*L. cuprina*, alimentary canal	Csak O:3	4	NDXE00000000
Ls15g	*C. sakazakii*	*Lucilia sericata*, blowfly, alimentary canal	Csak O:1	256	NDXF00000000

*All the assemblies along with the PGAP (Haft et al., 2018) annotations were released under FDA GenomeTrakr Bioproject on NCBI (PRJNA258403) and the genomic characteristics can be found in Supplementary Table 1. These isolates were obtained from flies by using the method described by Pava-Ripoll et al. (2012), Pava-Ripoll et al. (2015b). These strains were identified to their Cronobacter species identification using species-specific rpoB and cgcA PCR assays as described by Stoop et al. (2009); Lehner et al. (2012), and Carter et al. (2013). Sequence type designations for each strain were determined by uploading whole genome assemblies or PCR amplicon sequences (Baldwin et al., 2009) to the Cronobacter MLST website (http://pubmlst.org/cronobacter/, last accessed, 5/7/2020) and to the CFSAN GalaxyTrakR tool (https://galaxytrakr.org/, last accessed 5/7/2020) which scans genomes against PUBMLST schemes. UKN means that the serotype was not determined by PCR analysis. *^#^Abbreviations “s” or “g” refer to anatomical fly location that the isolate was obtained from; “s” refers to “surface” and “g” refers to “gut” or the fly’s “alimentary canal.”*

### Bacterial Strains: Identification and Molecular Characterization

Nineteen *Cronobacter* fly strains were characterized biochemically and identified according to the classification scheme defined by Iversen et al. (2008) and Joseph et al. (2012a), and their species identities were confirmed using both the *rpoB* and *cgcA* species-specific PCR assays as described by Stoop et al. (2009), Lehner et al. (2012), and Carter et al. (2013). Assistance in assigning *Cronobacter* species identity to these strains was carried out using a Gram-negative card analyzed with the Vitek-2 Compact platform (Biomerieux, Hazelwood, MO, United States). The Vitek-2 Compact instrument’s 5.03 software version was used to taxonomically place these strains into the *Cronobacter* species complex using a slash-line protocol analogous to that used for the identification of *Escherichia coli, Salmonella* spp., and other enteric Gram-negative bacterial pathogens. Identity of the strains as *Cronobacter* was further carried out by confirming the presence of the 350-bp amplified region of the genus-specific zinc metalloprotease (*zpx*) gene and finding the presence of species-specific plasmid gene targets and serotyping PCR assays (Kothary et al., 2007; Mullane et al., 2008; Franco et al., 2011a; Jarvis et al., 2011, 2013; Yan et al., 2015a). These strains were compared with strains obtained from clinical, food, and environmental sources which were characterized in previous studies (Franco et al., 2011a; Jarvis et al., 2011, 2013; Yan et al., 2015a,b; Chase et al., 2017; Gopinath et al., 2018; Jang et al., 2018a,b). Multiple locus sequence typing (MLST) of the strains was performed either by submitting genome sequences to the *Cronobacter* MLST website^1^ (last accessed 5/7/2020) or by performing the PCR reactions (to amplify the following genes: *atpD, fusA, glnS, gltB, gyrB, infB*, and *pps*) for sequencing according to the procedure described by Baldwin et al. (2009). Primer sequences and PCR reaction parameters can be found at the *Cronobacter* MLST website at https://pubmlst.org/cronobacter/info/protocol.shtml. For these strains, PCR amplicons were first purified using the Qiagen PCR purification kit (Qiagen, Inc. Germantown, MD), and submitted to Macrogen (Rockville, MD, United States) for sequencing. Sequence FASTA files were then uploaded to the *Cronobacter* MLST website for analysis. All genomes were also submitted to Center for Food Safety and Applied Nutrition’s (CFSAN’s) Galaxy GenomeTrakr website’s MLST tool (last accessed 5/7/2020) for analysis. Similarily, FASTA formatted genome assemblies were uploaded into CFSAN’s Galaxy GenomeTrakr AMRFinderPlus tool^2^ (last accessed, 5/7/2020) to scan each genome using a combined protein BLAST and hidden Markov model approach against a high-quality curated antimicrobial resistance (AMR) gene reference database which is designed to identify acquired antimicrobial resistance genes in bacterial AMR protein sequences as well as known point mutations for several taxa. More details can be found at https://github.com/ncbi/amr/wiki (last accessed 5/7/2020) (Zankari et al., 2012, 2017; Feldgarden et al., 2019). *C. sakazakii* strain 505108 co-harbors three plasmids of different incompatibility classes: IncHI2, IncX3, and IncFIB, respectively (Shi et al., 2018). p505108-MDR [National Center for Biotechnology Information (NCBI) accession #: KY978628] and p505108-NDM (New Delhi metallo-beta-lactamase 1, NCBI accession #: KY978629) FASTA files were downloaded from NCBI and used as the positive control for determining antimicrobial resistance genes in the fly strains. The antibiotics that the CFSAN Galaxy’s AMRFinderPlus tool identifies includes: aminoglycosides; amoxicillin/clavulanate (2:1); ampicillin; chloramphenicol; colistin; cefotaxime; cefoxitin; florfenicol; fusidic acid; gentamicin; macrolides; nitroimidazole; penicillin; quinolone; rifampicin; sulfamethoxazole; spectinomycin; streptomycin; tetracycline; trimethoprim; beta-lactams and extended spectrum beta-lactams (ESBLs), and ceftiofur.

To determine the presence and distribution of γ1a fimbriae among these *Cronobacter* strains, PCR primers were used. PCR primers shown in Table 2 were designed to target species-specific regions of the γ1a fimbriae chaperon-usher (CU) sequences. For amplification of γ1a CU genes in *C universalis, C. dublinensis, C. sakazakii*, and *C. muytjensii*, each species-specific forward PCR primer (γ1a_1215f, γa_1325f, γ1a_1529f, or γ1a_1621f, respectively) was mixed with primer γ1a_1830r. For amplification of the γ1a CU genes in *C. turicensis* and *C. malonaticus*, each species-specific reverse primer (γ1a_485r or γ1a_613r, respectively) was mixed with primer γ1a_220f. All primers used in the PCR amplification experiments were synthesized by Integrated DNA Technologies (Coralville, IA, United States). All PCR mixtures were prepared using the GoTaq Green master mix (Promega Corp., Madison, WI, United States) in a final reaction volume of 25-μl with 1 unit of GoTaq Hotstart DNA polymerase, 1.5 mM MgCl2, and 200 μM each deoxynucleoside triphosphate. Primers were added at 1 μM each, along with 1 μl DNA template (approximately 90 ng DNA/25-μl reaction mixture). In all PCRs, the polymerase was activated by using a 3-min incubation step at 94°C, followed by 30-cycles of denaturation at 94°C for 30 s, annealing temperature of 65°C for 15 s and an extension step at 72°C for 45 s. For each reaction, a final extension step of 5 min at 72°C completed the reaction cycle.

**TABLE 2:** PCR primers and amplicon size for determining presence of γ1a fimbriae gene in *Cronobacter* species.

Primer name	Sequence	Amplicon size (bp)	Species targeted
γ1a_1215f	TGCGATGGGGGCAGACGCTTA	616	*C. universalis*
γ1a_1325f	ACAACACGACCTCTACCGGCCA	506	*C. dublinensis*
γ1a_1529f	GTCAGACGCTCGACAGCATGGG	302	*C. sakazakii*
γ1a_1621f	GGCTTTGCCTGTACGACATCCGG	210	*C. muytjensii*
γ1a_1830r	GTCATCCATCAGSGTGCCGCT		*C. muytjensii-sakazakii-dublinensis-universalis*
γ1a_485r	CGCCTGCGGGATGCTTACCT	266	*C. turicensis*
γ1a_613r	GCGAGGAGTCCCCCTCACCT	394	*C. malonaticus*
γ1a_220f	GGCAACTACCCGCTGCGCAT		*C. turicensis- malonaticus*

### Preparation of Genomic DNA

All strains were grown overnight in a shaker incubator (160 rpm) at 37°C in 5 ml of Trypticase soy broth (BBL, Becton Dickinson, Franklin Lakes, NJ, United States) supplemented with 1% NaCl (final conc.). Genomic DNA was isolated from 2 ml of the culture with the robotic QIAcube workstation and its automated Qiagen DNeasy technology (QIAGEN Sciences, Germantown, MD, United States) following the manufacturer’s recommendations. Generally, 5–50 μg of purified genomic DNA was recovered in a final elution volume of 200 μl and used as a DNA template for PCR analysis. For microarray analysis, the purified DNA was then further concentrated using an Amicon Ultracel-30 membrane filter (30,000 molecular weight cutoff, 0.5 ml, Millipore Corp., Billerica, MA, United States) to a final volume of 10–25 μl as described by Tall et al. (2015).

### Microarray Design and Hybridization

The microarray used in this study is an Affymetrix MyGeneChip Custom Array (Affymetrix design number: FDACRONOa520845F) which utilizes the whole genome sequences of 15 *Cronobacter* strains, as well as 18 plasmids as described by Tall et al. (2015). Each gene (19,287 *Cronobacter* gene targets) was represented on the array by 22 unique 25-mer oligonucleotide probes, as described by Jackson et al. (2011) and Tall et al. (2015). Genomic DNA from *Cronobacter* and phylogenetically related species as listed in Supplementary Table 7 was hybridized, washed in the Affymetrix FS-450 fluidics station, and scanned on the Affymetrix GeneChip Scanner 3000 (AGCC software) as described by Jackson et al. (2011) and as modified by Tall et al. (2015). All reagents for hybridization, staining and washing were made in conjunction with the Affymetrix GeneChip Expression Analysis Technical Manual.

### Microarray Data Analysis, Calculating Gene Differences, and Generating Dendrograms

For each gene represented on the microarray, probe set intensities were summarized using the Robust MultiArray Averaging (RMA) function in the Affymetrix package of R-Bioconductor as described by Bolstad et al. (2003). RMA summarization, normalization, and polishing was done on the data and final probe set values were determined as explained by Jackson et al. (2011) and as modified by Tall et al. (2015). Gene differences were determined and phylogenetic trees were created using the SplitsTree 4 neighbor net joining method (Jackson et al., 2011; Tall et al., 2015). In a similar fashion, the distribution and prevalence of fimbriae genes were also determined using just fimbriae alleles which are captured on the FDA *Cronobacter* microarray and these alleles are shown in Supplementary Table 3.

### Whole Genome Sequencing and Nucleotide Sequence Accession Numbers

Whole genome sequencing (WGS) of the *Cronobacter* filth fly strains was performed using the MiSeq platform (Illumina, San Diego, CA, United States) with a Nextera XT library kit. Trimmed Fastq data sets were trimmed and *de novo* assembled with CLC Genomics Workbench version 9.0 (CLC bio, Aarhus, Denmark) as described by Grim et al. (2015) and Chase et al. (2016). All assemblies, along with the PGAP annotations (Haft et al., 2018), were released under FDA GenomeTrakr Bioproject on NCBI (PRJNA258403) which is part of FDA’s foodborne pathogen research umbrella project at NCBI (PRJNA186875). Genomic information regarding the strains are shown in Supplementary Table 1 which also includes genome accession numbers.

### Genome Sequence Annotation and Analysis

Genomes were annotated initially by using the SEED server (Overbeek et al., 2014) for developing this manuscript. A new core genome analysis workflow was developed using Spine software (Ozer et al., 2014). Briefly, filth fly and representative *Cronobacter* species genomes in FASTA format and their respective annotations in Genbank format were downloaded from NCBI. The server running at http://vfsmspineagent.fsm.northwestern.edu/cgi-bin/spine.cgi (last accessed 5/7/2020) was used for the analysis. A series of homology parameters were used to titer optimal configurations and are available upon request. The *C. condimenti* genome was defined as an outlier due to its genomic distance from the other *Cronobacter* species (Grim et al., 2013). *C. sakazakii* BAA-894 coding genes were used as the top-level reference for the analysis. The final Spine-derived ‘backbone sequences’ were designated as the ‘whole genome core-genes’ for studying phylogenetic relationship among fly isolates belonging to different *Cronobacter* species. NCBI standalone BLAST database suite was used for finding homologs. Briefly, a BLAST database of the filth fly strain genomes was created. Nucleotide sequences of putative virulence-associated protein coding gene loci from *C. sakazakii* BAA-894 was used to query this database. In-house Perl and Python scripts were used to process the data files (a default 90% nucleotide identity was used). Filth fly *Cronobacter* strain data sets were uploaded to the PHASTER (Phage Search Tool Enhanced Release) web server and pipeline^3^ (last accessed 5/7/2020) for phage sequence identifications (Zhou et al., 2011; Arndt et al., 2016).

### Zebrafish Infection Studies

To understand the virulence of these filth fly strains compared to other strains including those of clinical origins, zebrafish infection studies were performed. Husbandry, breeding and microinjection of approximately 50 CFU of bacteria into the yolk sac of 2 days post fertilization albino zebrafish (*Danio rerio*) was maintained following the original procedure described in the study by Fehr et al. (2015) and as updated by Eshwar et al. (2016). Virulence was assessed by determination of the survival rate (30 embryos: 10 embryos per bacterial strain, three independent experiments) over 72 hpi (=3 dpi). The number of dead embryos was determined visually based on the absence of heartbeat. Zebrafish husbandry and manipulation were conducted with approval (Licence Number 150) from the Veterinary Office, Public Health Department, Canton of Zurich (Switzerland). For experiments with zebrafish embryos and larvae ≤5 days post fertilization (dpf), no approval for animal experimentation is required according to the Swiss animal experimentation law. For statistical analysis, Kaplan–Meier survival analysis and statistics [Log-rank (Mantel–Cox) test] for zebrafish embryos infection experiments was performed with GraphPad Prism 7 (GraphPad Software, CA, United States). One-way ANOVA with *post hoc* Tukey HSD tests were used to assess the statistical significance.

## Results and Discussion

### Identification and AMR Characterization of *Cronobacter* Species Isolated From Filth Flies

During the surveillance study reported by Pava-Ripoll et al. (2012), 19 *Cronobacter* strains were obtained from filth flies from four fly species belonging to the families *Muscidae, Calliphoridae, Sarcophagidae*, and *Anthomyiidae* (one fly was identified only to the family level). Results are shown in Table 1. Two *C. malonaticus*, three *C. turicensis* and 14 *C. sakazakii* were identified from the alimentary canals and external surfaces of the filth flies. Interestingly, on five occasions, *Cronobacter* strains were isolated from both the alimentary canal (strain name labeled with suffix “g”) and the external body surface (strain name labeled with suffix “s”) of the same fly. In three of these instances, a fly was found that carried the same *Cronobacter* species at both anatomical sites: a red-tailed flesh fly carried *C. turicensis* serogroup unknown, ST569 strains (Sh41g and Sh41s), a house fly carried *C*. *sakazakii* serogroup O:1, ST8 strains (Md33g and Md33s), and a blowfly carried *C. sakazakii* serogroup O:3, ST4 strains (Lc10s and Lc10g). Another housefly carried a mixture of *C*. *sakazakii* ST4 strains differing only by serogroup designation: on its surface a *C. sakazakii* serogroup O:3 (Md5s), and in its alimentary canal a *C. sakazakii* serogroup O:2 strain (Md5g). Lastly, a *C. turicensis* ST546 strain (Md1sN, CturO:Unknown) was obtained from the body surface of another housefly and Md1g, a *C. sakazakii* serogroup O:2, ST4 strain was obtained from its alimentary canal. Two other houseflies carried *C. malonaticus* ST60 and ST7 strains (Md99g and Md25g, serogroups Cmal O:1 and Cmal O:2, respectively) in their alimentary canals. In addition to the *C. malonaticus, C. turicensis*, and *C. sakazakii* strains described above, there were seven other *C. sakazakii* isolated from filth flies that were identified as a ST93 *C. sakazakii* serogroup unknown (Md27gN), and two *C. sakazakii* ST8: one (Md35s) possessing serogroup CsakO:Unknown and the other (Md40g) possessed a CsakO:2 serogroup determinants. Additional *C. sakazakii* strains were found associated with these flies and include two ST4 *C. sakazakii* strains: (Md6g, Md70g), a ST221 strain (Ath48g), and a ST256 (Ls15g) possessing CsakO:3, CsakO:2, CsakO:Unknown, and CsakO:1 serogroup determinants, respectively. Taken together, MLST analysis of these *Cronobacter* strains (shown in Table 1) demonstrated that there were nine different STs found among the filth fly strains.

FASTA formatted files of plasmids p505108-multidrug resistant (MDR, NCBI accession #: KY978628.1) and p505108-NDM (New Delhi metallo-β-lactamase 1, NCBI accession #: KY978629.1) reported by Shi et al. (2018) were downloaded from NCBI. The corresponding *C. sakazakii* 505108 (ST1) sequences were used as positive control sequences, for comparison with the filth fly strains using the CFSAN AMRFinderPlus tool. The *bla*_*CSA/CMA*_ family of class C β-lactamase resistance genes described earlier by Müller et al. (2014) was possessed by all strains. This family of β-lactamases (AmpC) are non-inducible and are considered to be cephalosporinases. The results of this analysis are shown in Supplementary Table 2 and demonstrate that the *C. turicensis* and the *C. malonaticus* carried a *C. malonaticus* (CMA) class C *bla*_*CMA*_ resistance gene and that the filth fly *C. sakazakii* carried a *C. sakazakii* CSA class C *bla*_*CSA*_ resistance gene. Six of these CSA class C *bla* resistance genes were only identified to the family level whereas the remainding class C *bla* resistance genes were identified as either CSA-2 or CSA-1 variants. Additionally, the three *C. turicensis* strains, MD1sN, Sh41s, and Sh41g also possessed a *fosA* family fosfomycin resistance glutathione transferase gene. In contrast, CFSAN’s Galaxy’s AMRFinderPlus tool correctly identified all antimicrobial resistence/tolerance genes present on p505108-MDR (KY978628) which included the following antimicrobial resistance/tolerance genes: *bla_TEM–__1_, dfrA19, aph(3′′)-Ib, aph(6)-Id, mcr-9.1, aph(3′)-Ia, catA2, tet(D), aac(6′)-Ib3, bla_*SHV–*__12_, sul1, bla_*DHA–*__1_, qnrB4, qacEdelta1, arr, aac(3)-II, and aac(6′)-IIc* with predicted resistances/tolerances to β-Lactam based compounds, Trimethoprim, Aminoglycoside, Colistin, Phenicol, Tetracycline, Sulfonamide, Quinolone, Quaternary Ammonium, and Rifamycin antibiotics, respectively. The antimicrobial resistance genes found on plasmid p505108-NDM (KY978629) were identified as *ble, bla_*NDM–*__1_*, and *bla_SHV–__12_* with predicted resistances to bleomycin and NDM-1 β-Lactam antibiotics, consistent with previously results reported by Shi et al. (2018).

### Characterization of RepFIB Plasmids and pESA3- and pCTU1-Specific Gene Targets Among the Filth Fly Strains Compared to Other *Cronobacter* Strains

The common *Cronobacter* virulence plasmids, pESA3, CSK29544_1p, pCS2, pGW2, p1CFSAN068773, pSP291-1, pCMA1, p1, and pCTU1 share a high degree of sequence homology (Kucerova et al., 2010; Franco et al., 2011a; Stephan et al., 2011; Yan et al., 2013; Moine et al., 2016). Previously identified backbone genes or gene clusters which are known to be associated with these plasmids include a common incompatibility class IncF1B replicon (*repA*) and two iron (III) acquisition systems, *eitCBAD* (ABC heme transporter) and *iucABCD/iutA* (Cronobactin, a hydroxamate-type, aerobactin-like siderophore and the only known siderophore possessed by *Cronobacter*). Targeting of the shared *repA* gene and the two iron acquisition system gene clusters (*eitA* and *iucC* genes representing each gene cluster) by PCR showed that all 19 fly strains possessed *repA, eitA*, and *iucC* genes suggesting that the common virulence plasmids (pESA3-like, pCMA1-like or pCTU1-like) were possessed by these strains (Table 3). Furthermore, PCR analysis of these strains showed that 10 of the *C. sakazakii* were PCR-positive for the plasmid-borne Omptin-like protease, *Cronobacter* plasminogen activator gene (*cpa*), and four of these *C. sakazakii* strains were PCR-negative. The fly *C. malonaticus* and *C. turicensis* strains were also PCR-negative for this gene (Table 3). Franco et al. (2011b) showed that Cpa had significant identity to proteases belonging to the Pla subfamily of omptins such as PgtE which is expressed by *Salmonella enterica*, Pla of *Yersinia pestis*, and PlaA of *Erwinia* spp. They further showed that the proteolytic activity of Cpa allows for degradation of several host serum proteins, including circulating complement components, C3, C3a, and C4b. With degradation of these complement components by Cpa, it is thought that systemic circulating *Cronobacter* cells would then be protected from complement-dependent serum killing. Expression of Cpa was also found by Franco et al. (2011b) to inactivate the serum protein, plasmin inhibitor α2-antiplasmin (α2-AP) which leads to an unrestrained conversion of plasminogen to plasmin, further promoting systemic spread of these bacteria. Interestingly, *C. malonaticus, C. turicensis*, and all of the *cpa*-negative *C. sakazakii* (including Md33s, Md33g, Md35S, and Md40g) also lacked the conserved flanking regions surrounding *cpa* (Table 3). Interestingly, the *cpa*-PCR negative *C. sakazakii* strains were identified as ST8 strains (Table 1). Results showing that these *cpa*-PCR negative strains were also PCR-negative for the flanking regions (Δ*cpa*) strongly suggests that *cpa* is absent and the lack of amplification is not due to sequence variation that may have been associated with the PCR primer regions of *cpa*. Notably, Almajed and Forsythe (2016) reported similar findings for a ST8 clinical strain (*C. sakazakii* strain 680) isolated from an unknown cerebral spinal fluid sample from the United States, and these authors also reported that this strain was rapidly eliminated when incubated with human serum, despite possessing the plasmid. Additional representatives of *C. sakazakii* ST8 should be analyzed so as to determine whether or not this particular ST (and other related STs) has the propensity to be *cpa*-negative. Further research is also needed in order to better understand the molecular mechanisms of *cpa* expression in *Cronobacter*.

**TABLE 3:** Plasmidotype patterns* observed for the 19 *Cronobacter* strains isolated from filth fly.

Isolate	Species ID	pESA3/pCTU1 (incFIB)	*eitA*	*iucC*	*cpa*	Δ *cpa*	Δ T6SS	*T6SS IntL*	*vgrG*	*T6SS R end*	*T6SS IntR*	Δ fha	*fhaB*	*dfha*	pESA2/pCTU2 (incF2)	pCTU3 (incH1)
Md25g	*malonaticus*	(+)	(+)	(+)	(−)	(−)	(+)	(−)	(−)	(−)	(−)	(−)	(+)	(+)	(−)	(+)
Md99g	*malonaticus*	(+)	(+)	(+)	(−)	(−)	(+)	(−)	(−)	(−)	(−)	(−)	(+)	(−)	(−)	(−)
Sh41g	*turicensis*	(+)	(+)	(+)	(−)	(−)	(+)	(−)	(−)	(−)	(−)	(−)	(+)	(−)	(−)	(+)
Sh41s	*turicensis*	(+)	(+)	(+)	(−)	(−)	(+)	(−)	(+)	(−)	(−)	(−)	(+)	(−)	(−)	(+)
Md1sN	*turicensis*	(+)	(+)	(+)	(−)	(−)	(+)	(−)	(−)	(−)	(−)	(−)	(+)	(−)	(−)	(+)
Md1g	*sakazakii*	(+)	(+)	(+)	(+)	(−)	(−)	(+)	(−)	(−)	(−)	(+)	(−)	(−)	(−)	(+)
Md5s	*sakazakii*	(+)	(+)	(+)	(+)	(−)	(−)	(+)	(−)	(−)	(−)	(+)	(−)	(−)	(−)	(+)
Md5g	*sakazakii*	(+)	(+)	(+)	(+)	(−)	(−)	(+)	(−)	(−)	(−)	(+)	(−)	(−)	(−)	(+)
Md6g	*sakazakii*	(+)	(+)	(+)	(+)	(−)	(−)	(+)	(−)	(−)	(−)	(+)	(−)	(−)	(−)	(+)
Lc10s	*sakazakii*	(+)	(+)	(+)	(+)	(−)	(−)	(+)	(−)	(−)	(−)	(+)	(−)	(−)	(−)	(+)
Lc10g	*sakazakii*	(+)	(+)	(+)	(+)	(−)	(−)	(+)	(−)	(−)	(−)	(+)	(−)	(−)	(−)	(+)
Ls15g	*sakazakii*	(+)	(+)	(+)	(+)	(−)	(−)	(+)	(+)	(+)	(−)	(+)	(−)	(−)	(−)	(−)
Md27gN	*sakazakii*	(+)	(+)	(+)	(+)	(−)	(−)	(−)	(−)	(+)	(−)	(−)	(+)	(+)	(−)	(+)
Md33s	*sakazakii*	(+)	(+)	(+)	(−)	(−)	(−)	(−)	(+)	(−)	(−)	(−)	(−)	(−)	ND^#^	ND
Md33g	*sakazakii*	(+)	(+)	(+)	(−)	(−)	(−)	(−)	(−)	(−)	(−)	(−)	(−)	(−)	(−)	(+)
Md35s	*sakazakii*	(+)	(+)	(+)	(−)	(−)	(−)	(−)	(−)	(−)	(−)	(−)	(−)	(−)	(−)	(+)
Md40g	*sakazakii*	(+)	(+)	(+)	(−)	(−)	(−)	(−)	(−)	(−)	(−)	(−)	(−)	(−)	(−)	(+)
Anth48g	*sakazakii*	(+)	(+)	(+)	(+)	(−)	(−)	(+)	(+)	(+)	(−)	(−)	(+)	(+)	(−)	(−)
Md70g	*sakazakii*	(+)	(+)	(+)	(+)	(−)	(−)	(+)	(−)	(−)	(−)	(+)	(−)	(−)	(−)	(+)

*Results show whether the strains are PCR-positive or -negative using primers and PCR reaction parameters described by Franco et al. (2011a). ^#^ND is an abbreviation for Not Determined.

Also shown in Table 3 are the findings that all *C. malonaticus* and *C. turicensis* strains did not possess the T6SS gene cluster (ΔT6SS PCR-positive). Except for a single *C. turicensis* strain (Sh41s) which was PCR-positive for *vgrG, C. malonaticus* and *C. turicensis* strains also lacked, as expected, all T6SS gene cluster genes. Interestingly, four *C. sakazakii* ST8 strains (Md33s, Md33g, Md35g, and Md40g) also lacked the entire T6SS gene cluster. Interestingly, other *C. sakazakii* T6SS PCR-positive strains (e.g., Lc15g, Md27gN, and Anth48g) possessed most of the gene cluster (gene targets: T6SSIntL, *vgrG*, and T6SSRend). However, none of the filth fly *C. sakazakii* strains were PCR-positive for the T6SSIntR gene region. Taken together these results add support to the hypothesis that the plasmid-borne T6SS in *C. sakazakii* is in a state of “genetic flux” as posited by both Franco et al. (2011a) and Yan et al. (2015b). Recently, Wang et al. (2018) showed evidence of more than one T6SS gene cluster in *C. sakazakii* strain ATCC12868 that they denoted as T6SS-1 and T6SS-2. Their results suggest that T6SS-1 may contribute to interbacterial competition processes which may allow *C. sakazakii* to better compete with other species and the second gene cluster (T6SS-2) may be important during host interaction. They also proposed that the “genetic flux” seen in T6SS-2 gene cluster may lead to greater levels of systemic spread. However, why *C. sakazakii* strain ATCC12868 has multiple T6SS gene clusters contained within both the chromosome and plasmid pESA3 remains to be elucidated. Together, these results suggest that *C. sakazakii* may use these T6SS systems to quickly adapt to whatever stressful environment the bacterium encounters, e.g., environment versus host.

Lastly, the *C. malonaticus* and *C. turicensis* strains, as well as two of the *C. sakazakii* strains (Md27gN and Anth48g) possessed the filamentous hemagglutinin gene cluster as represented by the presence of *fhaB* (Table 3). The *fhaB-*positive *C. sakazakii* strains belonged to ST93 and ST221 (Table 1). According to the *Cronobacter* pubMLST site, both ST93 and ST221 are notably rare among sequence types described earlier for *C. sakazakii*. The finding that these strains possess *fhaB* is not surprising since Franco et al. (2011a) have previously reported that approximately 20% of *C. sakazakii* strains may possess *fhaB*. A comparison of the filth fly strain’s plasmidotyping results with that of other non-fly strains (data not shown) confirmed similar trends which were also observed previously by Franco et al. (2011a), and illustrate that no new plasmidotyping trends were identified at this time (Tall et al., 2019; Jang et al., 2020). Jang et al. (2018b) also investigated the prevalence and distribution of plasmid pESA3 genes associated with the virulence plasmids including pESA2 and pCTU3 harbored by 26 spice-associated *C. sakazakii*. They found that all of the strains possessed *eitA, iucC, cpa*, and the T6SS allele IntL but few possessed the rest of the T6SS gene cluster and six strains possessed the *fhaB* gene. However, it is interesting to speculate why certain *C. sakazakii* may possess this adhesin with such a high sequence homology to the filamentous hemagglutinin (FHA) of *Bordetella pertussis* (Franco et al., 2011a). Currently, it is not known if FHA is responsible for adaptive colonization of the upper respiratory tract and if it contributes to pneumonia cases such as those highlighted by Gosney et al. (2006), Patrick et al. (2014), and Alsonosi et al. (2015). This question remains to be explored and further research is needed to better understand the functional role of *fhaB* in *Cronobacter*.

To determine the presence of plasmids pESA2/pCTU2 and pCTU3 carried by the fly strains, we performed PCR analysis with these strains using pESA2/pCTU2-specific primers as described by Franco et al. (2011a). The results showed that none of the strains (only 18 strains tested in this study) possessed the conjugative pEAS2/pCTU2-like plasmids. Interestingly, *C. malonaticus* strain Md25g, 11 of the 19 *C. sakazakii* and all *C. turicensis* strains possessed the pCTU3-like plasmid (Table 3). pCTU3-like plasmid was originally described in *C. turicensis* by Stephan et al. (2011). These authors showed that this plasmid harbored genes responsible for arsenic and copper resistance, as well as silver ion transport. It is interesting to speculate as to why such fly strains possess a plasmid which harbors genes involved in heavy metal resistance via membrane-associated efflux transporters (Zhongyi et al., 2017; Negrete et al., 2019). The low prevalence of these plasmids is similar to that described recently by Tall et al. (2019) who had reported that pCTU3-like plasmids are found more often in *Cronobacter* than conjugative pCTU2/pESA2-like plasmids. It is currently unknown if flies benefit from having a population of bacteria living within their gut microflora which can express a heavy metal resistance phenotype or if maintaining such related genes or gene clusters has a role in heavy metal resistence in agriculture settings as suggested by Zhongyi et al. (2017). Interestingly, *C. sakazakii* ingested by adult houseflies was demonstrated to be transmitted to the fly progeny by Pava-Ripoll et al. (2015a), possibly by evolving several unknown mechanisms to evade the fly’s immune system. However, health benefits to filth flies provided by colonization of *C. sakazakii* still needs to be further studied.

### Pan Genomic Microarray Analysis Clustered the Fly Strains as Separate Species According to Sequence Type

**FIGURE 1:**
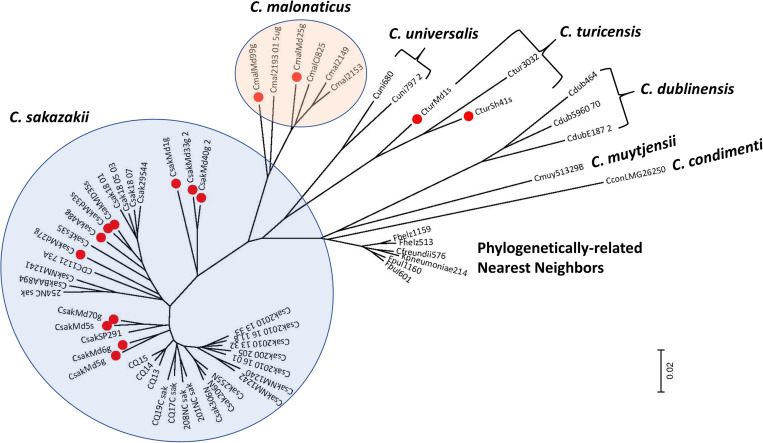
Phylogenetic analysis among 15 fly *Cronobacter* strains (identified with a red dot) and 45 other *Cronobacter* and phylogenetically-related strains was inferred using the Neighbor-Joining method using the 19,287 *Cronobacter* gene targets (Saitou and Nei, 1987) of the FDACRONOa520845F microarray. The optimal tree with the sum of branch length = 1.86949337 is shown. The tree is drawn to scale, with branch lengths in the same units as those of the evolutionary distances used to infer the phylogenetic tree. The evolutionary distances were computed using the p-distance method (Nei and Kumar, 2000) and are in the units of the number of base differences per site. The analysis involved 60 strains evaluated by using the FDA *Cronobacter* microarray. All positions containing gaps and missing data were eliminated. There were a total of 21,402 positions in the final dataset. Evolutionary analyses were conducted in MEGA7 (Kumar et al., 2016). These *Cronobacter* and phylogenetically related strains were analyzed from the presence-absence gene matrix (data not shown). The microarray experimental protocol as described by Jackson et al. (2011) and as modified by Tall et al. (2015) was used for the interrogation of the strains for this analysis. The phylogenetic tree illustrates that the *Cronobacter* microarray could clearly separate the seven species of *Cronobacter* with each species forming their own distinct cluster, and that representative fly strains clustered (identified with red dots) according to their species taxon. The scale bar represents a 0.02 base substitution per site.

To understand the phylogenetic relationship among the filth fly strains, DNA microarray hybridization experiments with 15 of the 19 strains were performed. Results were compared with other phylogenetically-related *Cronobacter* strains representing the seven *Cronobacter* species and nearest phlyogenetically-related taxa as listed in Supplementary Table 7. Take note that not all strains were tested by microarray analysis due to the cost of the each array. After applying the RMA-derived presence/absence gene algorithm, a gene-difference matrix was generated from the interrogation of these strains. From these data, as illustrated in Figure 1, a phylogenetic tree was generated which demonstrates that the microarray could accurately identify the filth fly strains to the correct *Cronobacter* species taxon. The filth fly strains grouped, as expected, with each of the three species clusters identified as *C. sakazakii, C. malonaticus*, and *C. turicensis*. As expected, the phylogenetic tree possessed a similar bidirectional genomic clustering characteristic to that previously described by Grim et al. (2013), Jackson et al. (2015), Killer et al. (2015), Tall et al. (2015), Chase et al. (2017), and Jang et al. (2018b) for other similar *Cronobacter* strains. An absolute correlation was found between the species identity of each filth fly strain analyzed by MA and that obtained by the validated end-point species-specific *rpoB* and *cgcA* PCR assays. Lastly and as expected, the *C. dublinensis* cluster was comprised of three separate clades which represented the three subspecies of *C. dublinensis* subsp: *dublinensis, lactaridi*, and *lausannensis*. Since only one strain of *C. muytjensii, C. universalis*, and *C. condimenti* was used in this analysis, these strains formed single species-strain clades which clustered separately. The lone *C. condimenti* strain was a distant outlier.

**FIGURE 2:**
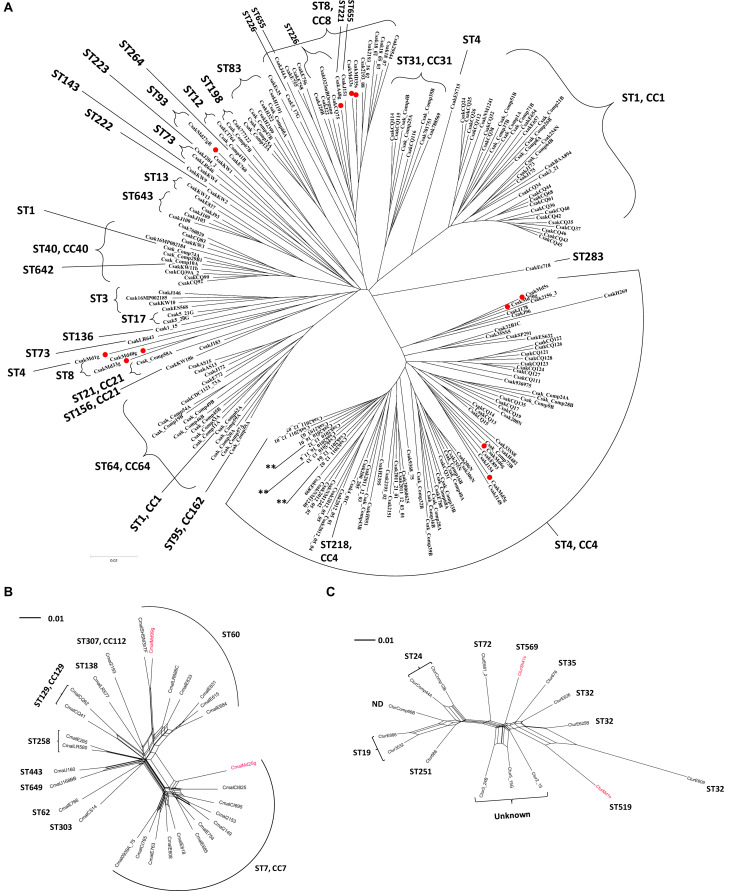
**(A)** Phylogenetic Neighbor net (SplitsTree4) analysis of representatives of *C. sakazakii* showing a bi-directional nature (along the tree *X*-axis). **(B)** Phylogenetic Neighbor net (SplitsTree4) analysis of representatives of *C. malonaticus.* The tree was generated from the gene-difference matrix (data not shown). **(C)** Phylogenetic Neighbor net (SplitsTree4) analysis of representatives of *C. turicensis* showing a bi-directional nature (along the tree *X*-axis). The tree was generated from the gene-difference matrix (data not shown). The microarray experimental protocol as described by Jackson et al. (2011) and as modified by Tall et al. (2015) was used for the interrogation of the strains for the analysis. The phylogenetic trees illustrate that the *Cronobacter* microarray could clearly separate these *Cronobacter* strains into distinct clusters based on ST phylogeny. Fly strains are identified by a red dot or red font. The scale bar represents a 0.01 base substitution per site. ND denotes not determined.

Figure 2A shows a phylogenetic tree developed for 11 filth fly *C. sakazakii* (red dots) in comparison with 195 other *C. sakazakii* strains. This figure represents a phylogenetic tree that was developed using microarray results and then overlaid with ST designations which were obtained using the seven allele MLST scheme as described by Baldwin et al. (2009) and CFSAN’s Galaxy MLST tool. As expected, the eleven *C. sakazakii* filth fly strains clustered according to ST designations. *C. sakazakii* strains Md33g and Md35g grouped together with other ST8 *C. sakazakii* strains [ST8, clonal complex (CC) 8 MLST profile: *atpD* 11, *fusA* 8, *glnS* 7, *gltB* 5, *gyrB* 8, *infB* 15, and *pps* 10] and filth fly strain Anth48g (ST221) also clustered close to these *C. sakazakii* ST8 strains, and as mentioned, *C. sakazakii* ST221 is a rare sequence type found in the *Cronobacter* MLST database. There is only one other strain (ID: 605; isolate name: CSWHPU-20; alias CY20) found in the database that possesses this ST designation (ST221 MLST profile: *atpD* 1, *fusA* 78, *glnS* 7, *gltB* 5, *gyrB* 8, *infB* 106, and *pps* 138), and which was isolated in 2013 in China from an infant noodle sample. To date and according to the *Cronobacter* MLST website, *C. sakazakii* strains possessing ST8, 111, 124 130, 133, 226, 279, 442, 608, 655 are members of the CC8 group. Interestingly, *C. sakazakii* Anth48g matches four of the seven alleles, but differs from these other ST8 strains by possessing the MLST alleles, *fusA* 78, *infB* 106, and *pps* 138, suggesting that ST221 strains may be related to ST8 strains and should to be considered as a member of *C. sakazakii* CC8 group. Interestingly, strain Md27gN which is a ST93 strain (ST93 MLST profile: *atpD* 15, *fusA* 1, *glnS* 3, *gltB* 32, *gyrB* 5, *infB* 36, and *pps* 190) clusters with another ST93 strain from the Republic of Korea. *C. sakazakii* filth fly strain Md1g (ST4), and ST8 strains Md40g and Md6g clustered together outside the main ST8 and ST4 clusters. Other strains which clustered outside their main ST clusters included ST1 strains Comp62A and Comp74A, and ST73 strain LR643. In summary, these results show that there is a good correlation between the seven allele sequence typing scheme and that obtained by MA. However, there were a few instances where the phylogenomic analysis based on the ∼19,000 *Cronobacter* genes gave a greater power of resolution when compared to the original seven allele MLST scheme. These results support that found in other studies as well (Chase et al., 2016; Gopinath et al., 2018; Jang et al., 2020).

Several points can be made regarding these findings using this combinatorial analysis approach. *C. sakazakii* filth fly strains [Md33s (ST8), Md35s (ST8), Anth48g (ST221), Md5g (ST4), Md5s (ST4), and Md70g (ST4)] grouped among strains of known corresponding ST and CC designations such as CC4 and 8, and these clusters were comprised of both clinically-relevant and PIF-associated strains. These results also suggest that filth fly ST4 and ST8 strains share genomic backbones similar to that of known clinical and food isolates of these respective STs (Joseph and Forsythe, 2011; Joseph et al., 2012b). For example, filth fly strains ST4 Md5g and Md70g clustered with two Jordanian strains cultured from spices, a clinical United States strain 2156 and strain H269, a PIF isolate from Switzerland. This combinatorial analysis approach also highlighted phylogenetic relatedness among groups of *C. sakazakii* strains with clonal complexes and ST designations that previously had not been recognized. For example, MA was able to show the phylogenetic relatedness among strains of several STs, e.g., ST8 strains clustering with strains of ST83, and ST1 strains clustering with ST31 strains, and ST13 strains clustering with strains of ST643 to name a few. However, at this point, caution is advised because of the small number of strains analyzed.

As shown in Figure 2B, the two *C. malonaticus* filth fly strains (red font) grouped among *C. malonaticus* strains possessing ST7 and ST60 which are known *C. malonaticus* sequence types of clinical importance. Filth fly strain Md25g clustered within the ST7/CC7 clade signifying that a close phylogenetic relatedness existed between this filth fly strain and other ST7/CC7 strains – many of which are from clinical sources – including the *C. malonaticus* type strain LMG 23826^*T*^ which was isolated from an adult with a breast abscess. The other filth fly strain, Md99g, clustered with ST60 *C. malonaticus* food associated and environmental strains.

A similar trend was also observed for the *C. turicensis* filth fly strains as shown in Figure 2C. The *C. turicensis* filth fly strains Md1sN and Sh41s (red font) were identified as an ST519 and ST569, respectively. Strain Sh41s (ST569) grouped as a singleton phylogenetically related to strains possessing ST designations of ST72 and ST35. Strain Md1sN grouped with three ST32 strains.

Taken together, this combinatorial phylogenetic analysis provides evidence that related strains from filth flies possess similar gene contents to that of strains from clinical, PIF, other foods, and man-made environmental sources, and that these pathotypes continue to circulate among the United States and European populations, environments, and filth flies. These results also suggest that there may be specific phylogenetically-related pathotypes which are circulating among *Cronobacter* populations that are not necessarily geographically distinct. This could very well be due to the present day global nature of the food supply. Such results argue for the need for greater vigilance of global food safety controls for the presence of these bacterial pathogens and their vector-associated fifth flies, and further comparative genomic investigations are warranted to understand these complex phylogenomic relationships. Finally, these data also further support the hypothesis that filth flies can serve as vectors of *Cronobacter* disease transmission.

### Presence of Fimbriae-Encoding Genes and Gene Clusters

Bacteria express filamentous assemblies of protein subunits on their exterior surfaces called adherence factors, pili or fimbriae which are used to colonize a host cell membrane surface or serve as conduits for the secretion of substrates, e.g., T4SS fimbriae. It is generally considered that bacterial adherence to an epithelial cell surface is the first step in pathogenesis (Finlay and Falkow, 1989). Adherence occurs either with the main structural fimbrial subunit or with associated fimbrial adhesins and are often target tissue specific. The genetic loci coding for these bacterial structures are found both on the chromosome and on plasmids (Thanassi et al., 2012).

Grim et al. (2013) identified eight fimbrial types among the seven *Cronobacter* species which was based on the chaperone-usher classification system described by Humphries et al. (2001). Grim et al. (2013) categorized these into a number of genomic regions (GR) encoding for γ1-fimbriae (in GR126 and GR82), genes for γ4-fimbriae (in GR52 and GR6), for κ-fimbriae (in GR112), β-fimbriae (in GR76), curli (in GR55), π-fimbriae (in GR9), and P-pilus homologs (in GR9, but missing in *C. sakazakii* strain BAA-894). Interestingly, these fimbriae types are also differentially dispersed among the *Cronobacter* genomes.

Some *Cronobacter* genomes also harbored curli biosynthesis genes, which are homologous to the curli of *Escherichia coli* and thin-aggregative fimbriae of *Salmonella* (Stephan et al., 2011; Hu, 2018). Curli fimbriae belong to a type of highly aggregated surface protein fibers (6–8 nm in diameter and 1 μm in length) classified as amyloids and are involved in adherence to cell or material surfaces, cell-cell aggregation, and biofilm development. The biosynthesis of curli is encoded by two operons, *csgBAC* and *csgDEFG* (csg, curli-specific genes in *E. coli*). Hu (2018), using primers designed to detect the structural curlin subunit gene (*csgA*) and a putative assembly factor gene (*csgG*), found that *csgA* could be identified in *C. dublinensis, C. malonaticus, C. turicensis*, and *C. universalis*, but not in *C. sakazakii* and *C. muytjensii*. Using the PATRIC tool (Wattam et al., 2017) and NCBI’s genome protein tables, the prevalence and distribution of type 1, β, Σ, Pap, and curli gene clusters possessed by the seven *Cronobacter* species were summarized by Jang et al. (2020). These authors presented evidence that curli biosynthesis genes are not found in *C. sakazakii* and *C. muytjensii*.

The FDA *Cronobacter* microarray contains probe sets representing fimbriae- or adhesin-related genes which were identified in genome regions (GR) described by Grim et al. (2013). β-fimbriae genes are represented on the microarray as 13 *C. sakazakii* genes that came from *C. sakazakii* strains Es15 (NCBI accession ID GCA_000263215) and BAA-894 (NCBI accession ID GCA_000017665) including seven genes of different β-fimbriae probable major orthologous subunits (corresponding to BAA-894 loci: ESA_03512, ESA_03513 and loci ESA_03517 to ESA_03521). The genes for the β-fimbriae chaperone and usher proteins correspond to BAA-894 loci ESA_03516 and ESA_03515. There are also seven β-fimbriae associated genes on the microarray from *C. dublinensis* subspecies *lactaridi*, but will not be discussed further because there was no cross hybridization with the filth fly strain samples probably due to the low protein percent sequence similarity (48.6–73.0%) with that of BAA-894. Interestingly and as shown in Supplementary Table 3, of the 13 genes representing β-fimbriae associated with *C. sakazakii, C. sakazakii* filth fly strain Md27gN hybridized with the β-fimbriae allele from *C. sakazakii* Es15 (Csak ES15.3466 corresponding to BAA-894 loci ESA_03512, ABU787727), and did not hybridize with the other six genes shared by *C. sakazakii* strain BAA-894. In contrast, all of the other *C. sakazakii* filth fly strains hybridized with the β-fimbriae probeset genes shared by *C. sakazakii* strain BAA-894 and did not hybridize with the β-fimbriae gene from *C. sakazakii* Es15. These results suggest that the filth fly *C. sakazakii* do possess β-fimbriae genes and that there are multiple β-fimbriae orthologous major subunit genes present in *C. sakazakii* genomes. All of the filth fly *C. sakazakii* hybridized with the β-fimbriae usher protein, ESA_03515 (ABU78730) and the β-fimbriae chaperone protein, ESA_03516 (ABU78731). As expected, the *C. malonaticus* and *C. turicensis* fly strains did not hybridize with these *C. sakazakii* genes.

A similar observation was also observed for genes representating type 1 fimbriae (Supplementary Table 3). Genes representing type 1 fimbriae-related genes on the microarray are from three *C. sakazakii* strains (BAA-894, 2151, and Es35), *C. malonaticus* LMG 23826^*T*^ and *C. turicensis* LMG 23827^*T*^. They are annotated as anchoring protein FimD (five *fimD C. sakazakii* orthologous genes ESA_02540, ESA_02343, ESA_01974, ESA_02797, and ESA_04071); fimbrial adaptor subunit FimG (ESA_02795 and ESA_02542; fimbrial adhesin precursor, ESA_02541; and chaperone protein FimC precursor, ESA_04072). Other type 1 fimbriae genes that are captured on the array include five *fimD C. sakazakii* orthologous genes (ESA_04071, ESA_02540, ESA_02343, ESA_01974, and ESA_02797), four *C. malonaticus fimD* genes (ESA_02343, ESA_02540, and ESA_04071), and three *C. turicensis fimD* genes (ESA_02343, ESA_02540, and ESA_04071). Also as expected, the microarray captured type 1 fimbrial genes from *C. condimenti, C. universalis, C. dublinensis* and *C. muytjensii* and that did not cross hybridize with any of the filth fly *C. sakazakii, C. malonaticus*, and *C. turicensis* strains.

Σ-fimbriae related genes are represented on the microarray by two genes from *C. sakazakii* strain BAA-894 (Σ-fimbriae chaperone protein, ESA_01345 and Σ-fimbriae tip adhesin, ESA_01347), two orthologs from *C. malonaticus* (Σ-fimbriae chaperone protein, ESA_01345 and Σ-fimbriae tip adhesin, ESA_01347), and three orthologs each from *C. turicensis* (Σ-fimbriae chaperone protein, ESA_01345; Σ-fimbriae usher protein, ESA_01346; and Σ-fimbriae tip adhesin, ESA_01347), and two copies of a Σ-fimbriae uncharacterized paralogous subunit, ESA_01344 and ESA_01343. Only filth fly *C. sakazakii, C. malonaticus*, and *C. turicensis* strains hybridized with each of the species-specific Σ- fimbriae genes. As expected, genes from the other *Cronobacter* species did not hybridize with these probes (Supplementary Table 3).

Genes for curli specific genes (*csg*) that are captured on the microarray are derived from *C. condimenti, C. dublinensis*, and *C. turicensis* (Supplementary Table 3). Additionally, there are several orthologs of a curlin transcriptional activator gene from *C. sakazakii* Es15 and *C. muytjensii* 51329^*T*^ (both ESA_03113). Eight of the filth fly *C. sakazakii* strains and *C. turicensis* strain Sh41g cross hybridized with this transcriptional activator gene from *C. sakazakii* Es15 and filth fly strains *C. turicensis* Md1sN and Sh41s hybridized with the transcriptional activator gene from *C. turicensis*. Other curli genes from *C. turicensis* z3032 include genes for curli assembly/transport (*csgG, csgE*, and *cggF*), for nucleation (*csgB*), the major curlin subunit (*csgA*), and a gene for a putative curli production protein (*csgC*). The two *C. malonaticus* fly strains did hybridize with *csgG, csgE, csgA*, but not *csgB* suggesting that these strains can secrete the curlin structural monomer, but may not nucleate the protein. All three fly *C. turicensis* strains did not hybridize with any of the curli assembly, nucleation, or subunit genes. These findings were corroborated by BLAST analysis of the WGS assemblies.

**FIGURE 3:**
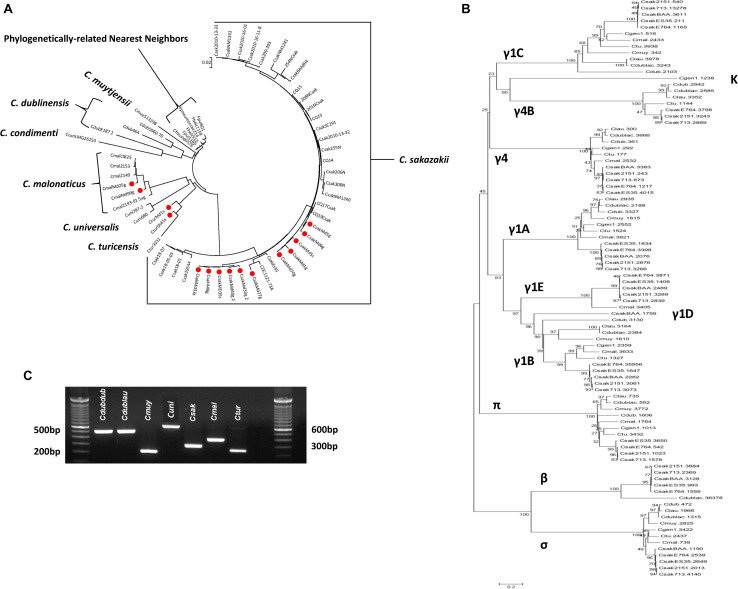
**(A)** Phylogenetic analysis among 15 fly *Cronobacter* strains (identified with a red dot) and 45 other *Cronobacter* and phylogenetically-related strains was inferred using the Neighbor-Joining method using only 355 fimbriae alleles that are represented on the FDA *Cronobacter* FDACRONOa520845F microarray (Saitou and Nei, 1987). The optimal tree with the sum of branch length = 1.22584234 is shown. The circular tree is drawn to scale, with branch lengths in the same units as those of the evolutionary distances used to infer the phylogenetic tree. The evolutionary distances were computed using the p-distance method (Nei and Kumar, 2000) and are in the units of the number of base differences per site. The analysis involved 60 strains. All positions containing gaps and missing data were eliminated. There were a total of 336 positions in the final dataset. Evolutionary analyses were conducted in MEGA7 (Kumar et al., 2016). The microarray experimental protocol as described by Jackson et al. (2011) and as modified by Tall et al. (2015) was used for the interrogation of the strains for the analysis. The scale bar represents a 0.02 base substitution per site. **(B)** Chaperon-usher tree developed using alignments of the sequences. The scale bar represents a 0.2 base substitution per site. **(C)** PCR analysis of *Cronobacter* spp. using PCR primers which target the γ-1a usher-chaperon region. Lanes 1–10 from left to right represent bar markers, *C. dublinensis* subspecies *dublinensis* LMG 23823, *C. dublinensis* subspecies *lausannensis* LMG 23824, *C. muytjensii* ATCC 51329, *C. universalis* NCTC 9529, *C. sakazakii* BAA-894, *C. malonaticus* LMG 23826, *C. turicensis* LMG 23827, empty lane, and bar markers.

Figure 3A shows the relationship among the filth fly strains which were analyzed with the microarray and the identities of various fimbriae gene clusters among the strains. The *Cronobacter* microarray contains 51 different usher-chaperone genes from all seven species and some species possessed genes of more than one fimbriae class. Interestingly, each *Cronobacter* species possessed species-specific fimbriae gene orthologs such that phylogenetically, MA using just fimbriae genes alone showed that each of the filth fly species clustered according to respective species groups. This analysis was extended to 242 *Cronobacter* strains and is shown in Figure 3B where a similar species-specific phylogeny trend was observed. Together, these results suggest that each *Cronobacter* species may possess common fimbrial orthologs and that the fimbriae sequence divergence has evolved along species lines. These findings are also supported by examining Supplementary Table 3 which shows the hybridization results (presence or absence) for fimbriae genes from *C. sakazakii, C. malonaticus*, and *C. turicensis* which are captured on the microarray. This analysis also illustrates that some of the probes cross hybridized among all three species with the pilus biogenesis *pilQ* gene possessed by *C. sakazakii* strain E764. This gene encodes for a membrane protein which may serve as a pore for the exit of the conjugation pilus but also as a channel for entry of heme and antimicrobial agents and uptake of transforming DNA. These findings were supported by the development of a preliminary species-specific PCR assay designed to identify *Cronobacter* species based on single nucleotide differences associated with the γ1a fimbriae class usher gene as shown in Figure 3C.

### Presence of Phage and Prophage Genes

**FIGURE 4:**
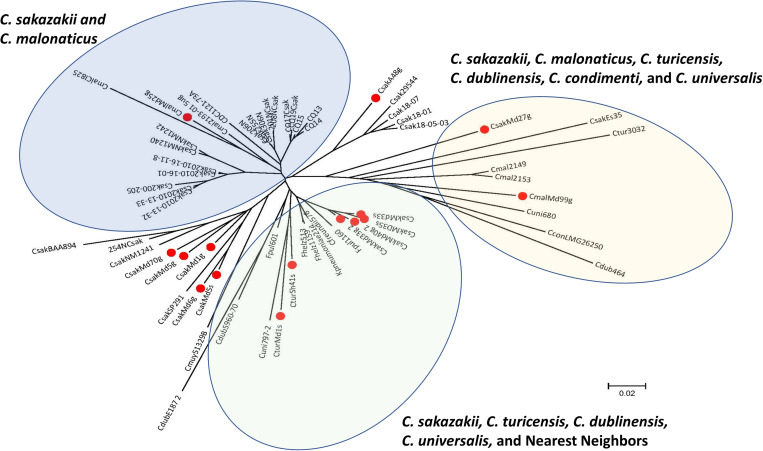
Phylogenetic analysis among 15 fly *Cronobacter* strains (identified with a red dot) and 45 other *Cronobacter* and phylogenetically-related strains was inferred using the Neighbor-Joining method using only 336 phage related alleles that are represented on the FDA Cronobacter FDACRONOa520845F microarray (Saitou and Nei, 1987). The optimal tree with the sum of branch length = 2.31200965 is shown. The tree is drawn to scale, with branch lengths in the same units as those of the evolutionary distances used to infer the phylogenetic tree. The evolutionary distances were computed using the p-distance method (Nei and Kumar, 2000) and are in the units of the number of base differences per site. The analysis involved 60 strains. All positions containing gaps and missing data were eliminated. There were a total of 655 positions in the final dataset. Phylogenetic analyses were conducted in MEGA7 (Kumar et al., 2016, 2018). The scale bar represents a 0.02 base substitution per site.

Figure 4 shows the relationship among filth fly strains analyzed with the microarray and the presence of phage- and prophage-related genes which are represented on the microarray. In contrast with the results of MA of the fimbriae-related genes shown in Figure 3A, the phage-associated genes were not species-specific in that interspecies clustering was observed among the *Cronobacter* species. The lack of species-specificity is not surprising given the promiscuity of phages. Nonetheless, though a phage-related species-specificity is not supported, the microarray can determine which phage genes are present in each strain. The microarray could not, however, determine if the presence of a phage in a particular strain is due to an active lysogenic event, or whether a phage is from a previous association that has lost its ability to be transmissible. More research is needed to further develop this hypothesis. Also, the fact that many *Cronobacter* phage can infect multiple species suggests that the surface structures used by the phage to infect cells are common among *Cronobacter*.

Filth fly and *C. sakazakii* BAA-894 FASTA data sets were uploaded to the PHASTER web server and pipeline to identify phage genomic regions. One hundred and fourteen incomplete, questionable, and intact phage gene encoding regions were found and this information is described in Supplementary Table 4. Fifty of these 114 sequences were identified as being intact and contained genes encoding for noted phage components for tail, plate, capsid, head, portal, terminase, lysin, recombinase, and integrase proteins. Total intact phage operons ranged from 15.4 to 143.8 kbp in size and encoded for 19–186 total proteins. There were three different intact *Cronobacter* phage identified in 7 of the 19 filth fly stains and included phage ENT39118_NC_019934, ENT47670_NC_019927, and phiES15_NC_018454 in strains Anth48, Md1g, Md1sN, Md27gN, Md5g, Sh41g, and Sh41s. One other *Cronobacter* phage was found as an incomplete phage gene cluster, CsaM_GAP32_NC_019401 in strains Md1g and Md5g. The diversity of intact phage found among the filth fly strains include three instances of phiO18P (NC_009542), a phage that comes from *Aeromonas media* in fly strains: Lc10g, Md5s, and Md6g. Another four instances of an intact *Edwardsiella* phage GF-2 (NC_026611) was found in fly strains: Lc10s, Md33g, Md35s, and Md40g. Three different generalized transducing Enterobacterial phages [ES18 (NC_006949), mEp235 (NC_019708), and 186 (NC_001317)] were found intact in three different filth fly strains Anth48g, Md25g, and Md99g. Of note *C. malonaticus* Md99g possessed both mEp235 and phage 186. An intact *Haemophilus influenzae* phage, HP-2 (HP2_NC_003315) was found in filth fly *C. turicensis* Md1sN. Also an intact *Mannheimia haemolytica* phage, vB_MhM_3927AP2 (NC_028766) was found in fly strains: Lc10g, Lc10s, Md5s, Md6g. There were four different *Salmonella* phages identified in the 13 filth fly strain genomes. Of those identified there were 25 intact phages and these included ten intact SSU5 (NC_018843) phage in strains Anth48, Lc10g, Lc10s, Md1g, Md25g, Md27gN, Md5g, Md5s, Md6g, and Md70g. There were 12 intact 118970_sal3 (NC_031940) identified in Lc10g, Lc10s, Ls15g, Md1g, Md5g, Md5s, Md6g, Md70g, and Md99g. There was one SPN3UB (NC_019545) phage identified as intact in Md1sN, and SPN9CC (NC_017985) was identified as intact in strain Md25g. In total, phages from eight different bacterial species were identified in addition to the four *Cronobacter* phages. Furthermore, finding such diversity of phages infecting *Cronobacter* strains supports the phylogenetic diversity found by MA.

### Presence of Hemolysin and Hemolysin-Related Genes

The gene encoding a hemolysin (*hly*) which may function in virulence was identified as a hemolysin III homolog (COG1272) by Cruz et al. (2011). Since then, several investigators have predicted that all *Cronobacter* species may possess a hemolysin III homolog (COG1272) gene (Kucerova et al., 2010; Grim et al., 2013; Moine et al., 2016). However, studies by Cruz et al. (2011), using a limited number of strains identified to the species level by using 16S RNA gene sequences and primers designed to target the gene, showed that some strains possessed the gene while others did not. Recently, Singh et al. (2017) described β-hemolytic activity in a limited number of *C. sakazakii* cultured from food, soil, and water; these strains were PCR-positive for the hemolysin III COG1272 allele. To better appreciate the gene prevalence and distribution, and phylogenetic relatedness of hemolysin III COG1272, we performed PCR analysis using the primers described by Cruz et al. (2011) on 390 *Cronobacter* strains of all species which were identified by species-specific PCR assays described by Stoop et al. (2009), Lehner et al. (2012), and Carter et al. (2013) and the results are shown in Table 4. PCR analysis demonstrated that hemolysin III COG1272 was present in 287/297 *C. sakazakii*; 3/7 *C. muytjensii;* 13/33 *C. malonaticus;* 3/12 *C. turicensis*; 3/36 *C. dublinensis*; and 1/1 *C. universalis*, respectively. *C. condimenti* was PCR-negative for hemolysin III COG1272. Parallel WGS analysis showed that all seven species do possess a hemolysin III COG1272 homolog but not necessarily present in all strains. Additionally, three other hemolysin genes were discovered by using this parallel next generation DNA sequence-based approach and these include genes encoding a cystathionine β-synthase (CBS) domain containing hemolysin, a putative hemolysin, and a 21-kDa hemolysin (Table 4 and Supplementary Table 3) and microarray analysis of the filth fly strains as well as certain *Cronobacter* species type strains showed that the filth fly *C. malonaticus* strains possessed the COG1272 hemolysin II homolog and cross hybridized with the COG1272 hemolysin II homolog from *C. sakazakii* BAA-894 and several of the other *C. sakazakii* filth fly strains such as Md5g, Md5s, Md6g, and Md70g and *C. turicensis* filth fly strain Sh41g. The COG1272 hemolysin homolog from BAA-894 cross hybridized with all of the filth fly *C. malonaticus* strains and all the *C. sakazakii* filth fly strains except for Md1g. Lastly, the probesets representing this gene from *C. condimenti, C. dublinensis, C. muytjensii* all hybridized with their respective donor strain. These results support the PCR findings that some strains of the *Cronobacter* species share this homolog. The 21-kDa hemolysin precursor from *C. turicensis* LMG 23827^*T*^ cross hybridized with *C. dublinensis* 5960-70, *C. malonaticus* LMG 23826^*T*^, six of the 12 *C. sakazakii* filth fly strains, and all of the *C. turicensis* filth fly strains as well as the two *C. universalis* analyzed. There were three probesets representing a hemolysin and related proteins containing a CBS domain gene from *C. condimenti, C. dublinensis*, and *C. turicensis*. The results showed that the *C. condimenti* probeset only hybridized with *C. condimenti* strain LMG 26250. The *C. dublinensis* probeset hybridized only with the *C. dublinensis* strains. However, the *C. turicensis* probeset hybridized with *C. turicensis* type stain LMG 23827^*T*^ and *C. turicensis* filth fly strain Sh41s, and cross hybridized with *C. sakazakii* filth fly strain Md27gN and *C. universalis* species type strain NCTC 9529^*T*^. The putative hemolysin allele was found intermittently among the strains that were analyzed. Umeda et al. (2017) recently reported that 57 *Cronobacter* strains exhibited β-hemolytic activity against guinea pig, horse and rabbit erythrocytes. However, using sheep erythrocytes, the majority of strains (53/57; 92.9%) showed α-hemolysis. Taken together, these results suggest that the primers designed by Cruz et al. (2011) may not detect hemolysin III COG1272 orthologs in all *Cronobacter* species uniformly and more in-depth genetic studies are needed to assign functionality of these various hemolysin genes to a hemolytic phenotype.

**TABLE 4:** Results of PCR analysis showing the prevalence and distribution of the hemolysin III COG1272 gene among 390 C*ronobacter* strains*.

Species	No. of strains PCR negative	No. of strains PCR positive	No. of strains tested	% positive
*C. sakazakii*	10	287	297	96.6
*C. muytjensii*	4	3	7	42.9
*C. malonaticus*	20	13	33	39.4
*C. turicensis*	9	3	12	25
*C. dublinensis*	33	3	36	8.3
*C. condimenti*	1	0	1	0
*C. universalis*	0	1	1	100
Total	79	311	390	79.7

*All strains were identified to their species epithet by using the species-specific PCR assays described by Stoop et al. (2009), Lehner et al. (2012), and Carter et al. (2013). Primer sequences and PCR reaction conditions were carried out according to Cruz et al. (2011). The hemolysin III COG 1272 gene ID in BAA-894 is ESA_RS01960, alias ESA_00432, and its location spans between 409401 and 410054 bp. The amplicon size of the PCR product spans from 409290 to 410170 and is 880 bp in size.

### Genome-Wide Analysis of Virulence-Associated Genes

To date, the *Cronobacter* microarray contains 40 genes that are annotated as putative or virulence-associated factors and represent a mixture of orthologous and species-specific genes from all seven species. Distribution of the homologs of these virulence-associated factors evaluated by MA provided a highly comparable profile of these predicted genes. The presence-absence calls (by MA) for these genes are shown in Supplementary Table 3. Notable genes (and NCBI protein ID) include the virulence factor MviM (ABU77528/ESA_02279), virulence protein MsgA (ABU76496/ESA_01234), virulence factor VirK (ABU77707/ESA_02461), putative virulence effector protein (ABU77161/ESA_01907), and the virulence-associated protein vagC (AHB72624). For the most part, the distribution of putative homologs followed evolutionary species and ST lines. However, there are a few genes which were shared among the these species: included a gene encoding a virulence protein MsgA from *C. dublinensis* subspecies *lausannensis* (NCBI ID ABU76496/ESA_01234) that was shared with filth fly *C. sakazakii* Md5g and Md6g, and the *C. turicensis* filth fly strain Md1sN. Virulence factor VirK (ABU77707/ESA_02461) from species type strain *C. universalis* NCTC 9529^*T*^ is shared with *C. sakazakii* fly strains A48g, Md27gN, and Md35s. Virulence-associated protein vagC (AHB72624) from *C. turicensis* type strain LMG 23827^*T*^ is also present in the three *C. turicensis* fly strains, Md1sN, Sh41g, and Sh41s; as well as in *C. malonaticus* fly strain Md25g, *C. sakazakii* fly strains Md1g, Md33g, Md33s, Md35s, Md40g, and Md5g, which suggests that vagC is in other *Cronobacter* species besides *C. turicensis*. Virulence factor MviM from *C. turicensis* type strain LMG 23827^*T*^ was also identified in *C. turicensis* fly strains Md1sN and Sh41s and was also found in *C. sakazakii* fly strains Md35s, Md5g, Md6g and Md70g.

BLAST analysis of the fly strain genomes using nucleotide sequences from BAA-894 resulted in identifying the distribution of the homologs of 32 virulence-associated genes as presented in the Supplementary Table 5.

### Core Gene Analysis and Comparative Genomics

Spine analysis was carried out on the filth fly genomes and from six of the seven *Cronobacter* species with *C. sakazakii* BAA-894 as the reference at 85, 90, and 95% levels of homology. This resulted in identifying 3,329, 2,905, and 767 core genes, respectively (Data not shown). Presence of *C. condimenti* genes impacted the homology threshold significantly and the genome was treated as an outlier in the final analyses. Considering the genomic diversity of *Cronobacter* species, a 90% cutoff value was set for refining the whole genome core gene (wg-core) loci to yield ∼2,790 genes consistently present in *C. sakazakii, C. malonaticus*, and *C. turicensis* branches of the distichously formed genomic tree as described by Grim et al. (2013). A data matrix was generated with single nucleotide polymorphisms (SNPs) present in the 19 genomes from the seven species in these backbone loci by BLAST analysis (data not shown) which re-created the genome diversity patterns observed earlier (Grim et al., 2013; Stephan et al., 2014; Tall et al., 2015; Chase et al., 2016; Gopinath et al., 2018; Jang et al., 2020) by WGS and the pan-genome microarray. The top 2,000 genes with a higher mutation rate from this data matrix were chosen as the *Cronobacter* final backbone reference wg-core gene set for subsequent analyses.

**FIGURE 5:**
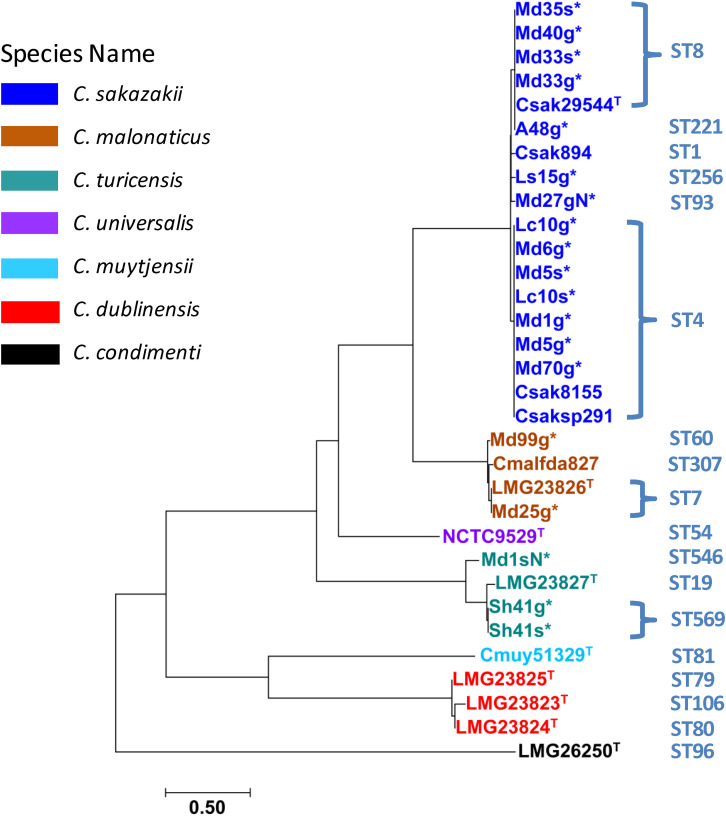
The phylogenetic analysis was inferred using the Neighbor-Joining method (Saitou and Nei, 1987). The optimal tree with the sum of branch length = 10.02357736 is shown. The tree is drawn to scale, with branch lengths in the same units as those of the evolutionary distances used to infer the phylogenetic tree. The evolutionary distances were computed using the Maximum Composite Likelihood method (Tamura et al., 2004) and are in the units of the number of base substitutions per site. The analysis involved 32 nucleotide sequences. All positions containing gaps and missing data were eliminated. There was a total of 308,989 positions in the final dataset. Evolutionary analyses were conducted in MEGA7 representing 2,000 core genes obtained from Spine analysis (Ozer et al., 2014; Kumar et al., 2016, 2018). The sequenced fly strains are labeled with an asterisk and the type strains are labeled with an upper case^*T*^.

Phylogenetic analysis of draft genomes from the fly strains were carried out by analyzing their genomes for SNPs specifically in these 2,000 reference core gene loci. The newly designed *Cronobacter* wg-core analysis clustered filth fly, reference and some clinical strains with a high resolution in a phylogenetic tree with manually curated MLST information added (Figure 5). Individual strain differences within some stains possessing similar STs were highlighted by this method. This analysis (Figure 5) demonstrates that ST4 *C. sakazakii* filth fly strains Lc10g, Lc10s, Md6g, Md5s, Md1g, Md5g, and Md70g clustered indistinguishably with a ST4 clinical strain *C. sakazakii* 8155 and a highly persistent Irish PIF manufacturing environmental strain SP291. Additionally, ST8 *C. sakazakii* O:1 fly strains Md35s, Md40g, Md33s, Md33g, and ST221 strain Anth48g clustered indistinguishably with ST8 *C. sakazakii* O:1 clinical strain ATCC 29544^*T*^. Similarly, ST7 *C. malonaticus* O:2 fly strain Md25g clustered indistinguishably with ST7 *C. malonaticus* O:2 clinical strain LMG 23826^*T*^ and strain Md99g clustered nearby to ST307 (CC112) *C. malonaticus* O:1 clinical strain 2193-01 (NCBI alias CmalFDA827). Lastly, ST569 *C. turicensis* strains Sh41g and Sh41s clustered indistinguishably with ST19 *C. turicensis* O:1 type strain LMG 23827^*T*^ and fly strain Md1sN clustered closely within this *C. turicensis* strain cluster.

Previously, whole genome SNP based clustering or phylogenetic analysis with conserved homologs of *C. sakazakii* BAA-894 with other species had been carried out by our group and others (Stephan et al., 2014; Tall et al., 2015; Chase et al., 2016, 2017; Gopinath et al., 2018; Jang et al., 2020). The new 2,000 wg-core gene reference loci data set developed as a powerful tool to accurately capture subtle differences in strains belonging to the same ST or ecological niche (as demonstrated in Figure 5). The complete list of 2,000 wg-core genes is provided in the Supplementary Table 6. This standardized genome-wide SNP finding tool provides the community with a method to query an ever-expanding repertoire of *Cronobacter* genome assemblies from different geographical areas and sources without any sample-bias, allowing the least ambiguity in SNP calls. For example, in Figure 5, it was noted that several of the ST4 *C. sakazakii* fly strains clustered indistinguishable with *C. sakazakii* strains 8155 and SP291 which are strains that were isolated from a can of dried milk in the 1950s and the environment of an Irish PIF manufacturing facility in 2014, respectively. These results suggest that the genomic-backbone of these isolates from very disparate sample sources (fly strains, dried milk, and PIF manufacturing environment) appear to be similar and highly conserved. Nevertheless, using the resulting data matrix from applying the 2,000 wg-core gene analysis, it is possible to extract SNP profiles in the wg-core loci shared by these two sets of isolates from exclusive sample sources for further analysis and methods development. Moreover, a robust dataset is generally needed for rigorous statistical analysis with bootstrap values to increase confidence in any phylogenetic analyses. Unlike traditional MLST methods that are susceptible to loss of resolution due to missing allelic information in the query dataset, our approach uses hundreds of conserved core genes for detecting phylogenetic features in a collection of isolates. As the populations of pathogens like *Cronobacter* are subject to varied evolutionary forces in different environments impacting genome sequences differentially will result in the emergence of smaller, but distinct clades. Results from studies reported by Chase et al. (2017) and Jang et al. (2018a, b) have reported sub-clades of *Cronobacter* isolates from new and unexplored environmental niches suggesting a genus with broader sequence variations than currently known. A standardized genome-wide tool that combines SNP data points with phylogenetic tree topologies and bootstrap support enhances better interpretation of WGS data (Pightling et al., 2018). The 2,000 wg-core gene schema presented here fills a critical gap for such a genome-wide analysis. This genome-wide approach was also applied to corroborate the overall similarity in the pattern of clustering as seen from pan-genomic microarrays in Figures 1–3.

### Zebrafish Infection Studies

Epidemiological studies of *in vitro* animal infection and mammalian tissue culture assays have shown that *Cronobacter* isolates demonstrate a variable virulence phenotype. To date, only isolates of *C. sakazakii, C. malonaticus*, and *C. turicensis* have been linked to infantile infections (Joseph et al., 2012b). However, adult infections far out number infantile infections, and epidemiologically the need for correctly identifying an isolate is paramount (Holý et al., 2014; Patrick et al., 2014; Alsonosi et al., 2015). Furthermore, comparative genomic and *in silico* analyses have proven to be powerful tools in elucidating potential virulence determinants in that the presence/absence of specific virulence determinants may explain the differential virulence behavior of strains (Franco et al., 2011a; Grim et al., 2013; Tall et al., 2015). Previously, the zebrafish embryo infection model confirmed the role of the RepF1B-like plasmids as virulence plasmids in *Cronobacter* and strengthened important roles for two virulence factors – *Cronobacter* plasminogen activator, *cpa* and the zinc-containing metalloprotease, *zpx* in pathogenesis (Eshwar et al., 2016).

**FIGURE 6:**
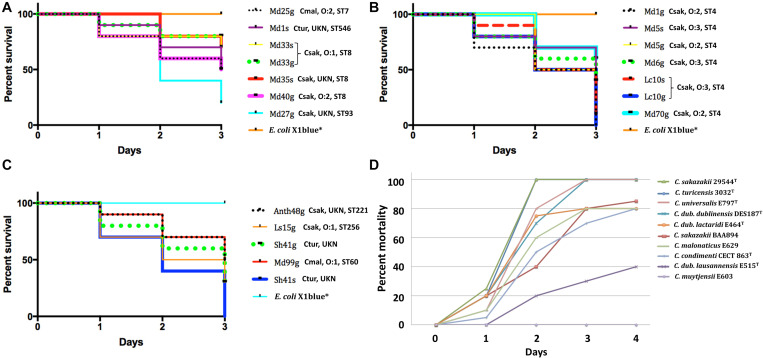
**(A–D)** Nineteen fly, other well characterized, and type species *Cronobacter* strains (strains identified as superscript T) were evaluated in the zebrafish embryo infection model as described by Eshwar et al. (2016). Experiments performed with *Cronobacter* strains involved the inoculation of the yolk sack of 30 embryos per strain. One-way ANOVA with *post hoc* Tukey HSD tests were used to assess the statistical significance. *p* = <0.0001. UKN indicates ‘unknown.’

Taking advantage of this newly described *Cronobacter* virulence model, we decided to access the virulence of the filth fly strains. Ten zebrafish embryos were infected with 50 CFUs of each of the 19 fly strains as well as a set of embryos infected with the negative control strain, *E. coli* strain Xl1blue as described by Eshwar et al. (2016). Due to a limited number of zebrafish, the strains that were evaluated were chosen because of species and ST. As shown in Figure 6, all of the filth fly strains caused between 25 and 100% mortality over an infection course of 3 days. Notably, strains Md1g (Csak O:2, ST4), Lc10g, Lc10s (Csak O:3, ST4), and Sh41s (Ctur, unknown ST) each had a mortality rate of 100% after 3 days post-injection. *C. malonaticus* strains Md99g (Cmal O:1, ST60) and Md25g (Cmal O:2, ST7) each caused a mortality rate of ∼50%. Zebrafish embryos infected with *E. coli* Xl1blue had a 100% survival rate, as expected and various *Cronobacter* species type strains gave variable results as expected and similar to that reported by Eshwar et al. (2016). Such results suggest that *Cronobacter* species harbored by flies have similar virulence attributes as that for known pathogenic strains that could lead to serious disease in human hosts. These results confirm flies as a viable host of pathogenic *Cronobacter* species.

## Conclusion

The primary focus of the research described in this study centered on using parallel next generation sequencing tools such as a pan genome-based DNA microarray, targeted PCR, and WGS analyses to subtype and specifically characterize *Cronobacter* strains which were isolated from filth flies and give a description of alleles that would help the scientific community focus on the development of methods based on these genomic features in future studies. This study provides results that further support the argument that flies can serve as vectors of *Cronobacter* species. The microarray has allowed a greater understanding of the divergence of species-specific genes and also has allowed for a greater understanding of the phylogenetic relatedness among STs and CCs not previously recognized. A proposal to augment existing ST phylogenetic relatedness schemes is proffered. Additional uses of the microarray would be the interrogation of additional strains within the clinical community which will help confirm or identify relevant virulence factors and lead to a better understanding of the epidemiology of the infections caused by these bacteria, and possibly shed light on the different pathotypes involved. This suggestion is supported by recent reviews (Farmer, 2015; Jason, 2015). However, one disadvantage of MA is that it will not capture unknown or new genetic attributes. A simplified phylogenetic method has been developed as part of this work using core genes in a limited number of representative genomes from each species of *Cronobacter*. Its application in phylogenetic analysis of filth fly strains was demonstrated. Putative homologs of selected virulence-associated loci identified in these vector-borne *Cronobacter* strains provides an opportunity for further research to study potential virulence properties of the members of the genus. Lastly, the corroborating data from parallel DNA microarray, WGS approaches, and virulence assessment of filth fly strains as described in this report, has led us to conclude that this scheme will be an undeniably powerful tool in identifying *Cronobacter* isolates by using the functional annotation already associated with the microarray along with the ability to capture, with WGS technology, unknown or new genetic attributes which together will protect public health against the threat of foodborne illness caused by *Cronobacter*.

## Data Availability Statement

The datasets generated in this study can be found in online repositories. The names of the repository/repositories and accession number(s) can be found in the article/Supplementary Material.

## Ethics Statement

This research was conducted with approval (NO 216/2012) from the Veterinary Office, Public Health Department, Canton of Zurich (Switzerland) allowing experiments with embryos and larvae older than 120 dpf and were carried out following the approved guidelines.

## Author Contributions

HJ, GG, BT, MP-R, AL, AE, SaF, and RS designed the study and finalized the submission. HJ, BT, GG, MP-R, SaF, CI, RS, JF, FP, SeF, FN, MK, LW, and AL wrote the initial and final drafts, respectively. AL and AE characterized and performed the zebra fish infections and analyzed the data. HJ, HC, SaF, FN, LW, and LC carried out genome sequencing, data submission, and the assembly and annotations. HC, QY, JG, IP, SJ, MM, and BT generated and analyzed the microarray data. GG carried out rest of the genomic analysis and illustrations. LW and FN carried out phage and plasmid analysis. All authors contributed to the article and approved the submitted version.

## Conflict of Interest

The authors declare that the research was conducted in the absence of any commercial or financial relationships that could be construed as a potential conflict of interest.

## Footnotes

1 http://pubmlst.org/cronobacter/

2 https://galaxytrakr.org/

3 https://phaster.ca/
