# Supplementary material for: Analysis of the Molecular Diversity Among Cronobacter Species Isolated From Filth Flies Using Targeted PCR, Pan Genomic DNA Microarray, and Whole Genome Sequencing Analyses
Source: Front Microbiol. 2020 Sep 25;11:561204. doi: 10.3389/fmicb.2020.561204 (PMC7545074; doi:10.3389/fmicb.2020.561204)
Supplement: Supplementary file 1 [file Table_1.PDF]

Supplementary Table 1. Genome characteristics of strains used in the comparative genomic analysis for selected fly *Cronobacter* strains

Biosample ID	Strain name	Genome size (bases)	No. of contigs	% G+C	No. of CDS	Sequence type (ST)	WGS GenBank Accession No.
MOD1_Md25g	Md25g	4665619	314	56.6	4319	7	MSAC00000000
MOD1_Md99g	Md99g	4424527	101	57.0	4089	60	MSAF00000000
MOD1_Sh41g	Sh41g	4636321	140	57.3	4242	569	MRZS00000000
MOD1_Sh41s	Sh41s	4635229	75	57.3	4242	569	MSAG00000000
MOD1_Md1sN	Md1sN	4354150	80	57.6	4001	519	VOEL00000000
MOD1_Md1g	Md1g	4688868	78	56.5	4367	4	MSAH00000000
MOD1_Md27gN	Md27gN	4646385	231	56.6	4300	93	VOEK00000000
MOD1_Md33g	Md33g	4448874	119	56.8	4149	8	MSAI00000000
MOD1_Md33s	Md33s	4467164	260	56.8	4138	8	MRXC00000000
MOD1_Md35s	Md35s	4454850	135	56.8	4144	8	MRXD00000000
MOD1_Md40g	Md40g	4456538	213	56.8	4139	8	MRXE00000000
MOD1_Md6g	Md6g	4709875	104	56.5	4419	4	MRXB00000000
MOD1_Md70g	Md70g	4592017	115	56.6	4298	4	MRXG00000000
MOD1_Anth48g	Anth48g	4544887	194	56.8	4170	221	MRXF00000000
MOD1_Md5s	Md5s	4708547	78	56.5	4417	4	MRWZ00000000
MOD1_Md5g	Md5g	4584917	120	56.6	4216	4	MRXA00000000
MOD1_Lc10s	Lc10s	4649493	153	56.6	4346	4	NDXD00000000
MOD1_Lc10g	Lc10g	4704855	158	56.5	4399	4	NDXE00000000
MOD1_Ls15g	Ls15g	4575958	126	56.8	4231	256	NDXF00000000
